# Comparison of Modified Mallampati Classification in Supine and Sitting Positions to Predict Difficult Tracheal Intubation in Diabetic Patients

**DOI:** 10.5812/aapm-145034

**Published:** 2024-04-07

**Authors:** Diya Sarah Jacob, Sonal Bhat, Sunil Vasudev Rao

**Affiliations:** 1 Junior Resident, Manipal, India; 2 Department of Anaesthesiology, Kasturba Medical College, Mangalore, Manipal Academy of Higher Education, Manipal, India

**Keywords:** Mallampati, Supine, Sitting, Diabetic, Difficult Intubation

## Abstract

**Background:**

Airway management of patients with long-standing diabetes poses a major challenge for anaesthesiologists due to stiff joint syndrome affecting the atlanto-occipital joint. In certain cases requiring immobilization, the Mallampati test must often be performed in the supine position for obvious reasons.

**Objectives:**

Hence, we determined the diagnostic precision (sensitivity and specificity) of the modified Mallampati test in sitting and supine positions among the diabetic population in predicting difficult tracheal intubation.

**Methods:**

A single-center prospective observational study on adult diabetic patients undergoing general anesthesia and orotracheal intubation was carried out. An observer recorded the modified Mallampati in the sitting posture during the pre-anesthetic examination. The Mallampati in the supine position was determined while in the operating room, and the difficulty of intubation was noted, and diagnostic precision was calculated. The main objective was to predict a difficult airway by calculating the sensitivity, specificity, positive predictive value, and negative predictive value.

**Results:**

Out of the 150 participants, Mallampati grading in a sitting position was correctly able to identify 42.5% of difficult intubation cases, whereas it was 97.5% with Mallampati in the supine position. Mallampati grading in the sitting position was able to correctly identify 89.1% of easy intubation cases, which was 63.6% with Mallampati in the supine position. The correlation of Mallampati in the supine position with CL grading was statistically significant (P < 0.001).

**Conclusions:**

Among diabetic patients, the modified Mallampati test in the supine position can be considered a more accurate and sensitive predictor of difficult intubation than the sitting posture.

## 1. Background

Difficult intubations remain a concern for anaesthesiologists and can be potentially fatal. The American Society of Anaesthesiologists (ASA) defines a difficult airway as any clinical situation in which anticipated or unanticipated difficulty or failure is experienced by a physician trained in anesthesia care, including but not limited to one or more of the following: Facemask ventilation, laryngoscopy, ventilation using a supraglottic airway, tracheal intubation, extubation, or invasive airway (1). Airway management of patients with long-standing diabetes poses a challenge for anaesthesiologists due to stiff joint syndrome affecting the atlanto-occipital joint. It limits adequate extension of the head and neck during laryngoscopy, leading to difficulty in intubation. The original and modified Mallampati tests are being used as simple bedside screening tests to detect difficult intubation (2). It is a simple scoring system that correlates the amount of mouth opening to the size of the tongue and provides an estimate of space available for oral intubation by direct laryngoscopy (3, 4). It was defined and studied with the patient sitting, head in the neutral position, mouth wide open, tongue protruding, and without phonation (3, 4). However, in certain cases requiring immobilization (like pain or neck injury), traumatic spine injuries, and fractures of the lower limb and hip, the test must often be performed in the supine position for obvious reasons. Furthermore, a lack of a standard method to evaluate the modified Mallampati classification limits interpretation and thereby produces conflicting results on the accuracy of the test (5). There is a paucity of studies on using these tests among the diabetic population to predict difficult intubation.

## 2. Objectives

Therefore, the aim of the study was to determine the diagnostic precision (sensitivity and specificity) of the modified Mallampati test in sitting and supine positions among the diabetic population.

## 3. Methods

A prospective observational study was conducted after obtaining approval from the Institutional Ethics Committee (IEC KMC MLR 06/2022/255) and Clinical trial registry of India (CTRI/2023/02/049398). Patients were selected by convenience sampling. The inclusion criteria were all individuals aged 18 and above with a history of type 2 diabetes mellitus of more than two years undergoing general anesthesia of ASA physical status II, III, IV, and were willing to give informed consent. Patients not willing to provide consent, pediatric patients, uncooperative patients, patients with a Glasgow Coma scale (GCS) less than 15, obstetric patients, patients with tumor masses in the oral cavity, immobile atlanto-occipital joint, maxillofacial trauma, large anterior neck mass, patients on long-term anti-inflammatory drugs, and patients undergoing regional anesthesia were excluded from the study. The sample size was calculated using MedCalc (ver. 20.110). Considering an alpha error of 5%, power of 80%, assuming the area under the curve to be 0.82 (supine) and 0.65 (sitting) for variables of interest in the present study, and keeping a ratio of easy vs. difficult tracheal intubation as 2, we needed a minimum sample size of 34 difficult tracheal intubation patients and 68 easy tracheal intubation patients for the present study. Therefore, a total of 102 diabetic patients would be required in the present study. The study protocol was explained to the patients, and written informed consent was obtained. Pre-operative airway assessment was done in sitting and supine positions using the modified Mallampati classification (MMC). The study was single-blinded, meaning the observer who assessed the Cormack Lehane score in the operating theatre was blinded to preoperative airway assessment. The study was conducted in teaching hospitals affiliated with Kasturba Medical College, Mangalore, Karnataka, from February 2023 to February 2024. Assessment in the sitting position was done with the head in a neutral position, mouth opened to the maximum, and tongue protruded maximally with the observer seated opposite the patient's eye level. While conducting the assessment in the supine position, the participant’s head was placed on a 10 cm high pillow, and the observer assessed the airway by looking vertically downward with the table height fixed at the observer’s hip level. It was categorized as (1) class I – visualization of the soft palate, fauces, uvula, and pillars; (2) class II – visualization of the soft palate, fauces, and uvula; (3) class III – visualization of the soft palate and base of the uvula; (4) class IV – soft palate not visible. In the operating room, the participants were pre-oxygenated, premedicated with Midazolam (0.02 mg/kg) and Fentanyl (2 – 3 mcg/kg), induced with Propofol (2 mg/kg). Neuromuscular blockade was administered with Atracurium (0.5 mg/kg)/ Vecuronium (0.1 mg/kg) after confirming the depth of anesthesia, and participants were ventilated with a bag and mask until adequate muscle relaxation was achieved.

Laryngoscopy was attempted by an anesthesiologist with more than 2 years of experience blinded to the MMC. All laryngoscopies were done using a metallic Macintosh blade, and the blade size was chosen according to the patient. During intubation, glottic exposure was graded using the Cormack – Lehane grading: (1) Grade I – Full view of the glottis; (2) Grade IIA – Partial view of the glottis, anterior commissure not visible; (3) Grade IIB – Only arytenoids seen; (4) Grade III – Only epiglottis seen; (5) Grade IV – Neither epiglottis nor glottis seen. Difficult Tracheal Intubation in the present study followed the definition: 'insertion of the endotracheal tube with conventional laryngoscopy requiring more than 2 attempts or lasting > 10 minutes, or requiring an alternate technique (bougie, video laryngoscope, fiberoptic)', and accordingly, tracheal intubation was classified as easy, difficult, and impossible. modified Mallampati classification in the sitting and supine positions was then compared to Cormack Lehane grading, and diagnostic accuracy (sensitivity, specificity, positive and negative predictive values, and positive and negative likelihood ratios) were calculated.

### 3.1. Statistical Analysis

The data were analyzed using SPSS for Windows (SPSS ver. 22.0, IBM Corp., Armonk, NY). Quantitative data were reported as mean and standard deviation, whereas qualitative data were reported in percentages. The chi-square test was used to associate categorical data with demographic factors like age, gender, and Body Mass Index. Data were presented as graphs and tables. Sensitivity, specificity, positive predictive value, negative predictive value, and diagnostic accuracy were calculated for MMC in the sitting and supine positions with CL grading, respectively. The level of significance was set at P ≤ 0.05.

## 4. Results

A total of 150 patients were enrolled, of which the majority of participants were in the age group of 51 – 70 years, and females outnumbered males. Fifty nine and three tenths percent of participants had a normal BMI, and 30% of participants were overweight. Table 1 shows that there was no association between the type of intubation and MMC grading in the supine position with age and gender (P > 0.05). However, it was found that difficult intubation using MMC grading in the supine position was statistically significantly more common among obese participants than normal participants (P = 0.001).

**Table 1.** Association Between Intubation as Assessed by Mallampati Grading in the Supine Position With Age, Gender, and Body Mass Index

**Variables**	**MMC Supine**		**P-Value ** ^a^
	Difficult, N (%)	Easy, N (%)	
**Age (y)**			0.34 ^NS^
< 40	8 (80)	2 (20)	
41 - 50	22 (57.9)	16 (42.1)	
51 - 60	19 (45.2)	23 (54.8)	
61 - 70	23 (50)	23 (50)	
> 70	7 (50)	7 (50)	
**Gender**			0.84 ^NS^
Males	31 (51.7)	29 (48.3)	
Females	48 (53.3)	42 (46.7)	
**BMI**			0.001
< 18	0	5 (100)	
19 - 24.9	40 (44.9)	49 (55.1)	
25 - 30	29 (64.4)	16 (35.6)	
> 30	10 (90.9)	1 (9.1)	

Abbreviations: NS, not significant using the chi-square test; N, number.

^a^Statistically significant at P < 0.05 and P < 0.01 using chi-Square test.

In the sitting position, 46.7% of participants belonged to the class 2 Mallampati category, followed by 34% in the class 1 category. In the supine position, 42.7% of participants belonged to the class 3 category, followed by 36% in the class 2 category. Forty two percent of participants belonged to Grade 1 Cormack Lehane, and 31.3% were categorized as Grade 2A Cormack Lehane. The least percentage and number of participants belonged to the Grade 4 category.

**Figure 1.**
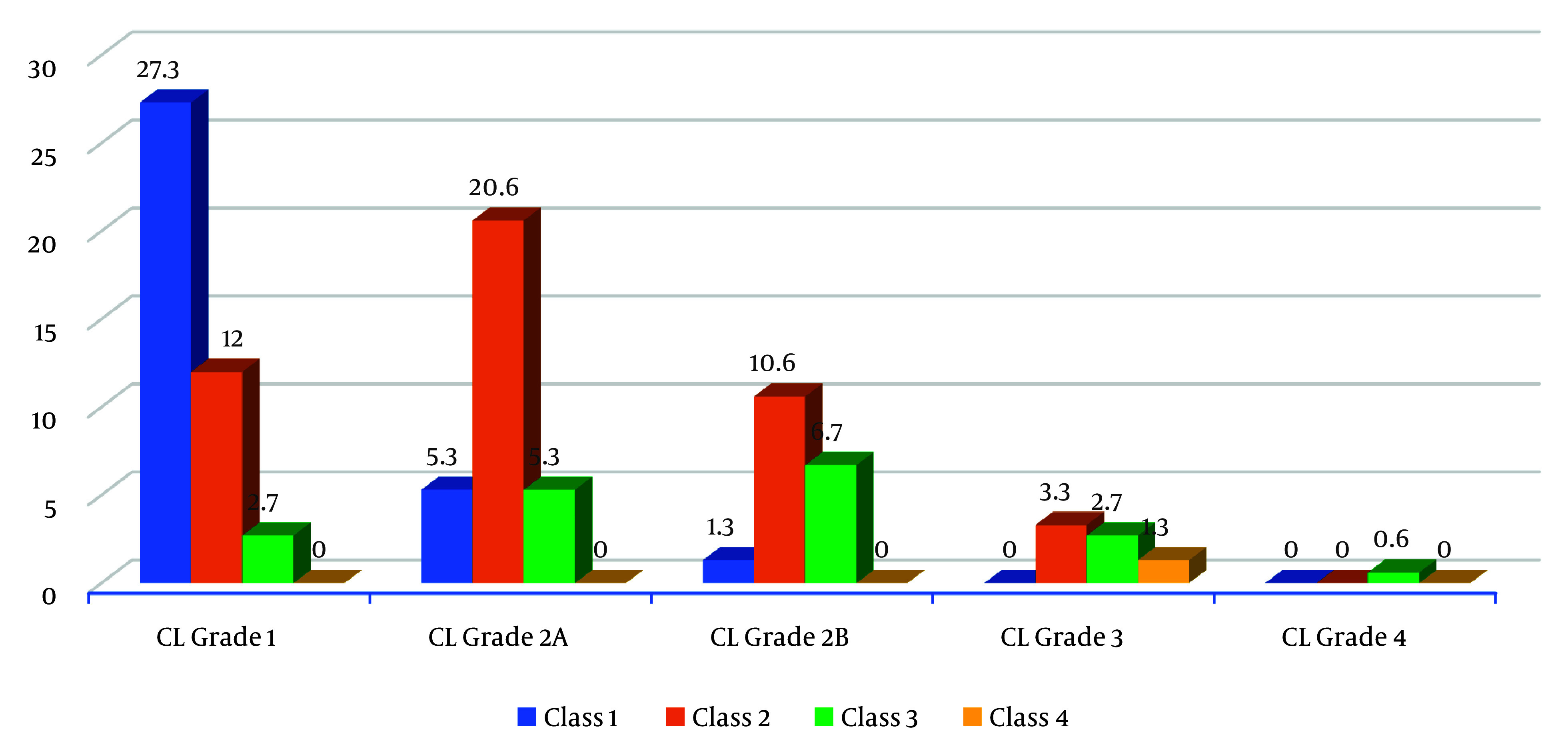
Distribution of participants according to Mallampati (sitting position) and Cormack-Lehane grading.

Table 2 and Figure 1 show that 27.3% of participants who had CL Grade 1 view of the larynx also had a Mallampati class I airway in the sitting position. Additionally, 20.6% of participants who had CL Grade 2A view of the larynx also had a Mallampati class 2 airway. About 10.6% of participants who had CL grade 2B view of the larynx had a Mallampati class 2 airway. This distribution was found to be statistically significant (P = 0.001).

**Table 2.** Distribution of Participants According to Mallampati (Sitting Position) and Cormack-Lehane Grading

	CL Grading					Total	P-Value
	**Grade 1**	**Grade 2a**	**Grade 2b**	**Grade 3**	**Grade 4**		
**MMC**	N (%)	N (%)	N (%)	N (%)	N (%)	N	0.001^a^
**Class 1**	41 (27.3)	8 (5.3)	2 (1.3)	0 (0)	0 (0)	51	
**Class 2**	18 (12)	31 (20.6)	16 (10.6)	5 (3.3)	0 (0)	70	
**Class 3**	4 (2.7)	8 (5.3)	10 (6.7)	4 (2.7)	1 (0.6)	27	
**Class 4**	0 (0)	0 (0)	0 (0)	2 (1.3)	0 (0)	2	
**Total**	63 (42)	47 (31.3)	28 (18.6)	11 (7.3)	1 (0.6)	150	

Abbreviations: N, number; MMC, modified mallampati classification.

^a^Statistically significant according to chi-square test.

**Figure 2.**
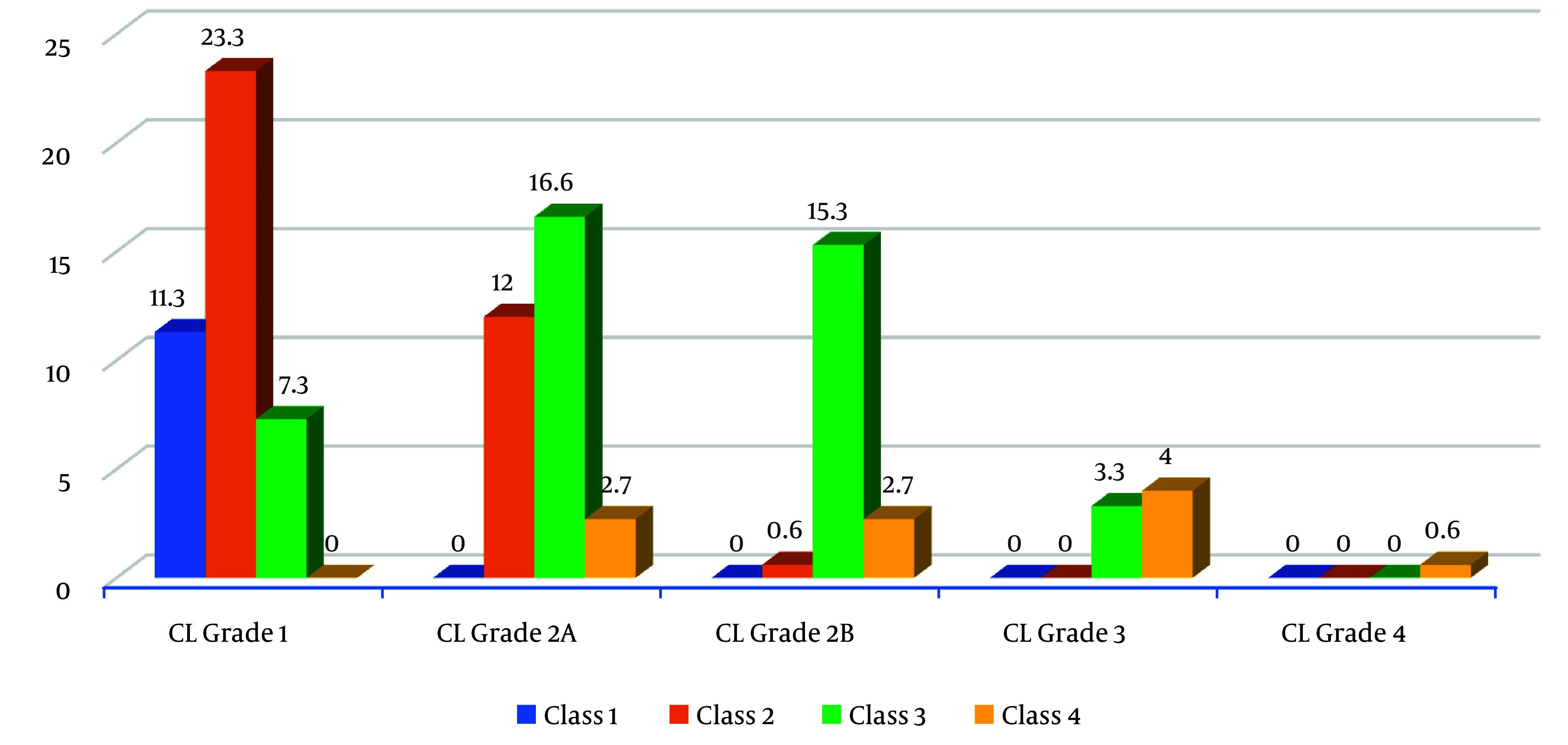
Distribution of participants according to Mallampati (supine position) and Cormack-Lehane grading

Table 3 and Figure 2 show that 23.3% of participants who had CL Grade 1 view of the larynx also had a Mallampati class 2 airway in the supine position. Additionally, 16.6% of participants who had CL Grade 2A view of the larynx also had a Mallampati class 3 airway. About 15.3% of participants who had CL grade 2B view of the larynx had a Mallampati class 3 airway. This distribution was found to be statistically significant (P = 0.001).

**Table 3.** Distribution of Participants According to Mallampati (Supine Position) and Cormack-Lehane Grading

Variables	Grade 1	Grade 2a	Grade 2b	Grade 3	Grade 4	Total	P-Value
**MMC**	N. (%)	N (%)	N (%)	N (%)	N (%)	N	P = 0.001^a^
**Class 1**	17 (11.3)	0 (0)	0 (0)	0 (0)	0 (0)	17	
**Class 2**	35 (23.3)	18 (12)	1(0.6)	0 (0)	0 (0)	54	
**Class 3**	11 (7.3)	25 (16.6)	23 (15.3)	5 (3.3)	0 (0)	64	
**Class 4**	0 (0)	4 (2.7)	4 (2.7)	6 (4)	1 (0.6)	15	
**Total**	63 (42)	47 (31.3)	28 (18.6)	11 (7.3)		150	

Abbreviations: N, number; MMC-modified mallampati classification.

^a^Statistically significant according to chi-square test.

Table 4 illustrates the distribution of participants categorized as difficult and easy intubation based on Mallampati Grading in both sitting and supine positions.

**Table 4.** Distribution of Participants as Difficult and Easy Intubation According to Mallampati Grading in the Sitting Position and in the Supine Position

Variables	MMC Supine		Total
**MMC sitting**	**Difficult**	**Easy**
**Difficult**	29	0	29
**Easy**	50	71	121
**Total**	79	71	150

Abbreviation: MMC, modified mallampati classification.

Table 5 reveals that Cormack-Lehane Grading with Mallampati grading in the sitting position correctly identified 42.5% of difficult intubation cases, whereas it reached 97.5% with Mallampati in the supine position. Additionally, Cormack-Lehane Grading with Mallampati grading in the sitting position correctly identified 89.1% of easy intubation cases, compared to 63.6% with Mallampati in the supine position. The Positive Predictive Value (PPV) for Cormack-Lehane Grading with Mallampati grading in the sitting position was 58.6%, while it was 49.4% with Mallampati in the supine position. The Negative Predictive Value (NPV) of Cormack-Lehane grading with Mallampati in the sitting position was 81%, contrasting with 98.6% with Mallampati in the supine position.

**Table 5.** Sensitivity, Specificity, Positive Predictive Value, Negative Predictive Value, and Accuracy of Cormack-Lehane Grading With Mallampati in the Sitting Position, Mallampati in The Supine Position, and Mallampati Grading in Both Sitting and Supine Positions

Variables	CL Grading with MMC (Sitting)	CL Grading with MMC (Supine)	MMC Sitting and Supine
**Sensitivity**	42.5	97.5	36.7
**Specificity**	89.1	63.6	100
**Accuracy**	76.7	72.7	66.7
**Prevalence**	26.7	26.7	52.7
**Positive predictive value**	58.6	49.4	100
**Negative predictive value**	81	98.6	58.7

Abbreviation: MMC, modified mallampati classification.

Furthermore, Mallampati grading in both sitting and supine positions correctly identified 36.7% of difficult cases and 100% of easy cases. The PPV of Mallampati grading in the sitting and supine positions was 100%, while NPV was 58.7%, respectively.

It was observed that difficult intubation using MMC grading in the supine position was statistically significantly more common among obese participants than normal participants (P = 0.001).

Moreover, difficult intubation was found to be statistically significantly more prevalent among female participants than male participants (P = 0.024). Additionally, difficult intubation using CL grading was statistically significantly more common among obese participants than normal participants (P = 0.001).

## 5. Discussion

The Mallampati test is routinely conducted in a sitting position to predict difficult airways, aiming to prevent potentially fatal airway complications if not managed promptly. It is believed that diabetic patients undergo non-enzymatic glycosylation and abnormal collagen deposition in joints, leading to stiff joint syndrome that can impact the atlanto-occipital joint (6, 7), making Diabetes Mellitus a predictor for difficult intubation (8). In this study, it was observed that the sitting posture detected 17 out of 40 true difficult intubation cases with a sensitivity of only 42.5%, while the supine position identified 39 out of 40 true difficult intubation cases with a sensitivity of 97.5%. Additionally, the specificity of the supine position was 63.6% due to a higher number of False Positive cases, while the sitting position demonstrated a specificity of 89.1%, indicating correct detection of 98 out of 110 easy intubation cases.

Gonadane et al. conducted a study among the diabetic population and found that Mallampati grading in the sitting position detected 21 out of 26 difficult cases with a sensitivity of 80.77% (9). In contrast, Thomas and Hashim reported that Mallampati grading could not predict difficult intubation and suggested that Palm Print was a better predictor (10). Mashour et al. reported a sensitivity of 41.2% and specificity of 76% among obese patients with Mallampati grading in the sitting position, also noting that diabetes is a predictor of difficult intubation among obese individuals (11). Compared to these findings, our study showed better sensitivity in the supine posture but contrasted with regards to the sitting posture.

To ascertain if the incidence of difficult intubation among the diabetic population is equivalent to non-diabetics, we attempted to compare these findings with the normal population. Our results align with Markos et al., who reported better sensitivity for supine posture (78.8% vs. 97.5%) but higher specificity for sitting posture (75% vs 42.5%), but sitting had better specificity (93%) (12). In contrast, Bindra et al., found sitting posture to have better sensitivity in predicting difficult intubation than the supine posture (13). Additionally, we found that the sitting posture correctly predicted 29 out of 79 difficult cases, albeit with a lower sensitivity of 36.7% and a specificity of 100%. This suggests that Mallampati classification in the sitting posture was a better diagnostic predictor for easy intubation cases compared to the supine posture.

However, Gondane et al. and Thomas and Hashim reported slightly lower specificities of 41.18% and 68.1% by Mallampati grading in the sitting position, respectively (9, 10). Baig and Khan reported a specificity of 99.2% among the diabetic population, which was comparable to our finding (14). Furthermore, the accuracy of Mallampati in both postures in predicting difficult intubation was calculated. The sitting posture exhibited higher accuracy than the supine posture (76.7% vs. 72.7%), which was lower compared to the accuracy reported by Baig & Khan (14) (92.2%) and higher compared to the accuracy reported by Thomas and Hashim (58.3%) (9). The PPV leaned more towards the sitting posture. The percentage of difficult intubations as a proportion of all difficult intubations in the sitting posture was 58.6%, whereas it was 49.4% for the supine posture. Among diabetic populations, George and Jacob in 2003 reported a PPV of 77.8% in the sitting posture (15). Similarly, the NPV favored the supine posture. The percentage of cases correctly predicted as not difficult intubations was 98.6% for the supine posture and 81% for the sitting posture. Additionally, the sitting posture exhibited a higher (100%) PPV and a lower NPV compared to the supine posture. The NPV from the present study was greater than the NPV from other studies reported in the literature. When comparing the NPV of diabetics with the normal population, the results were similar to a study conducted by Khatiwada et al. (16). The results were inconsistent with the results from studies conducted by Bindra et al. (12), who reported a higher PPV and NPV, respectively.

In the present study, about 33.3% of females were categorized as having difficult intubation under the Cormack-Lehane grading. Oria et al.(17)reported similar results, but studies conducted by Wang et al. (18) in 2019 reported difficult intubation to be significantly associated more with males than females. About 90.9% and 81.8% of obese participants were categorized as having difficult intubation in both supine posture and Cormack-Lehane grading, respectively. This indicates an association between high BMI and difficult intubation assessed using Mallampati and Cormack-Lehane grading. A Meta-analysis conducted by Wang et al. in 2018 concluded that obesity was associated with an increased risk of difficult intubation (18). We did not study the combination of MMC with phonation either in a sitting or supine position. Additionally, the anatomy of study participants might vary according to their ethnic backgrounds, and study participants in the present study were not stratified according to their ethnicity. Furthermore, a Receiver Operator Curve for MMC in supine and sitting posture would have provided more valid results.

## Data Availability

The dataset presented in the study is available on request from the corresponding author during submission or after publication. The data are not publicly available due to privacy and ethics.

## Clinical Trial Registration Code:

CTRI/2023/02/049398.
